# Circulating miRNAs as Novel Non-Invasive Biomarkers to Aid the Early Diagnosis of Suspicious Breast Lesions for Which Biopsy Is Recommended

**DOI:** 10.3390/cancers13164028

**Published:** 2021-08-10

**Authors:** Marta Giussani, Chiara Maura Ciniselli, Loris De Cecco, Mara Lecchi, Matteo Dugo, Chiara Gargiuli, Andrea Mariancini, Elisa Mancinelli, Giulia Cosentino, Silvia Veneroni, Biagio Paolini, Rosaria Orlandi, Massimiliano Gennaro, Marilena Valeria Iorio, Catherine Depretto, Claudio Ferranti, Gabriella Sozzi, Marialuisa Sensi, Mario Paolo Colombo, Gianfranco Scaperrotta, Elda Tagliabue, Paolo Verderio

**Affiliations:** 1 Laboratory Medicine Unit, Diagnostic Pathology and Laboratory Department, Fondazione IRCCS Istituto Nazionale dei Tumori, 20133 Milan, Italy; marta.giussani@istitutotumori.mi.it; 2 Bioinformatics and Biostatistics Unit, Department of Applied Research and Technological Development, Fondazione IRCCS Istituto Nazionale dei Tumori, 20133 Milan, Italy; chiara.ciniselli@istitutotumori.mi.it (C.M.C.); mara.lecchi@istitutotumori.mi.it (M.L.); paolo.verderio@istitutotumori.mi.it (P.V.); 3 Platform of Integrated Biology, Department of Applied Research and Technological Development, Fondazione IRCCS Istituto Nazionale dei Tumori, 20133 Milan, Italy; loris.dececco@istitutotumori.mi.it (L.D.C.); chiara.gargiuli@istitutotumori.mi.it (C.G.); marialiusa.sensi@istitutotumori.mi.it (M.S.); 4 Department of Medical Oncology, IRCCS Ospedale San Raffaele, 20132 Milan, Italy; dugo.matteo@hsr.it; 5 Department of Biomedical Sciences, Humanitas University, 20090 Pieve Emanuele, Italy; andrea.mariancini@humanitasresearch.it; 6 Chemical-Clinical and Microbiological Analyses, ASST Grande Ospedale Metropolitano Niguarda, 20162 Milan, Italy; elisa.mancinelli@ospedaleniguarda.it; 7 Molecular Targeting Unit, Department of Research, Fondazione IRCCS Istituto Nazionale dei Tumori, 20133 Milan, Italy; giulia.cosentino@istitutotumori.mi.it (G.C.); rosaria.orlandi@istitutotumori.mi.it (R.O.); marilena.iorio@istitutotumori.mi.it (M.V.I.); 8 Tissue Biobank, Department of Applied Research and Technological Development, Fondazione IRCCS Istituto Nazionale dei Tumori, 20133 Milan, Italy; silvia.veneroni@istitutotumori.mi.it; 9 Anatomic Pathology A Unit, Department of Diagnostic Pathology and Laboratory, Fondazione IRCCS Istituto Nazionale dei Tumori, 20133 Milan, Italy; biagio.paolini@istitutotumori.mi.it; 10 Breast Surgery Unit, Fondazione IRCCS Istituto Nazionale dei Tumori, 20133 Milan, Italy; massimiliano.gennaro@istitutotumori.mi.it; 11 Breast Unit, Imaging, Fondazione IRCCS Istituto Nazionale dei Tumori, 20133 Milan, Italy; Catherine.Depretto@istitutotumori.mi.it (C.D.); claudio.ferrantii@istitutotumori.mi.it (C.F.); Gianfranco.scaperrotta@istitutotumori.mi.it (G.S.); 12 Tumor Genomics Unit, Department of Research, Fondazione IRCCS Istituto Nazionale dei Tumori, 20133 Milan, Italy; gabriella.sozzi@istitutotumori.mi.it; 13 Molecular Immunology Unit, Department of Research, Fondazione IRCCS Istituto Nazionale dei Tumori, 20133 Milan, Italy; mario.colombo@istitutotumori.mi.it

**Keywords:** breast cancer, diagnosis, circulating biomarkers, microRNAs

## Abstract

**Simple Summary:**

In population-based screens, tissue biopsy remains the standard practice for women with imaging that suggests breast cancer. We examined circulating microRNAs as minimally invasive diagnostic biomarkers to discriminate malignant from benign breast lesions. A retrospective cohort of plasma samples divided into training and testing sets and a prospective cohort of women with suspicious imaging findings who underwent tissue biopsy were investigated through a global microRNA profile by OpenArray. Seven signatures, involving 5 specific miRNAs (miR-625, miR-423-5p, miR-370-3p, miR-181c, and miR-301b), were identified and validated in the testing set. Among the 7 signatures, the discriminatory performances of 5 of them were confirmed in the prospective cohort.

**Abstract:**

In population-based screens, tissue biopsy remains the standard practice for women with imaging that suggests breast cancer. We examined circulating microRNAs as minimally invasive diagnostic biomarkers to discriminate malignant from benign breast lesions. miRNAs were analyzed by OpenArray in a retrospective cohort of plasma samples including 100 patients with malignant (T), 89 benign disease (B), and 99 healthy donors (HD) divided into training and testing sets and a prospective cohort (BABE) of 289 women with suspicious imaging findings who underwent tissue biopsy. miRNAs associated with disease status were identified by univariate analysis and then combined into signatures by multivariate logistic regression models. By combining 16 miRNAs differentially expressed in the T vs. HD comparison, 26 signatures were also able to significantly discriminate T from B disease. Seven of them, involving 5 specific miRNAs (miR-625, miR-423-5p, miR-370-3p, miR-181c, and miR-301b), were statistically validated in the testing set. Among the 7 signatures, the discriminatory performances of 5 were confirmed in the prospective BABE Cohort. This study identified 5 circulating miRNAs that, properly combined, distinguish malignant from benign breast disease in women with a high likelihood of malignancy.

## 1. Introduction

Breast cancer (BC) is the most frequent cancer among women, and despite screens for its early diagnosis, it remains a leading cause of cancer-related mortality worldwide [1]. The Breast Imaging Report and Data System (BI-RADS) lexicon was introduced by the American College of Radiology to score the risk of suspected BC in imaging studies [2,3] and determine the need for image-guided biopsy. BI-RADS categories 4 and 5 classify suspicious lesions for which biopsy is recommended. However, while BI-RADS 5 findings are greatly suggestive of BC, BI-RADS 4 lesions are highly variable in the outcome group, having a probability of malignancy ranging from 3% to 95%. Thus, some patients have benign lesions but undergo unnecessary biopsies or, in some cases, surgery. Biopsy remains required to prove that suspicious imaging findings are malignant or benign in 7% to 10% of women who undergo breast cancer screens, as reported by the National Centre for screening monitoring (https://www.osservatorionazionalescreening.it/, accessed on 9 August 2021).

Tissue biopsy is an invasive procedure that represents a cost for the health system. Thus, a simple and minimally invasive test to overcome these drawbacks remains an unmet clinical need. One such option is to monitor circulating molecular markers in blood that distinguish benign from malignant breast disease. Over the last 20 years, the advent of “omics” strategies has led to novel approaches in the search for noninvasive biomarkers for diagnosing BC. Circulating carcinoma antigens, tumor cells, cell-free tumor DNA and RNA, and extracellular vesicles in the peripheral blood have appeared as potential biomarkers that supplement current clinical tools [4]. 

MiRNAs are a class of short noncoding, single-stranded RNAs that regulate gene expression at the post-transcriptional level by binding to target mRNAs. miRNAs are commonly dysregulated in various human cancers, becoming oncogenes or tumor suppressor genes and regulating several steps in neoplastic transformation (reviewed in [5,6]). Differences in miRNA expression in various malignancies have been examined primarily as biomarkers for the diagnosis, prognosis, and response to treatment in cancer. miRNAs can be secreted by several cell types into the extracellular space and then shuttled to peripheral blood in a form that is resistant to digestion by RNases through their encapsulation by extracellular vesicles or binding to lipoproteins. Because miRNAs are stable in routinely collected clinical liquid samples, in contrast to mRNA, these molecules constitute a class of reliable, minimally invasive cancer biomarkers that merit interest in the detection of early-onset disease (reviewed in [7]). 

Circulating miRNAs are indicative of BC [8,9,10,11,12,13,14,15,16,17,18,19], and the combination of certain circulating miRNAs distinguishes BC from normal and healthy controls [20,21,22,23,24]. However, benign breast lesions that may yield diagnostic images that indicate BC have rarely been included in these studies, and the number of samples that have been considered has been limited [25,26,27,28]. Thus, the development of an accurate and reliable panel of circulating miRNAs for the early diagnosis of BC in women with suspicious diagnostic images remains a challenge.

In this study, we attempted to discriminate malignant from benign breast disease by analyzing circulating miRNAs in a training set and a testing set of retrospectively collected plasma samples from BC patients, women with breast benign disease, and healthy donors and performing a prospective clinical study of women with suspicious imaging findings (BI-RADS 4–5) who underwent biopsy to obtain a correct histopathological diagnosis of malignant or benign disease.

## 2. Materials and Methods

### 2.1. Plasma Samples

**Figure 1:**
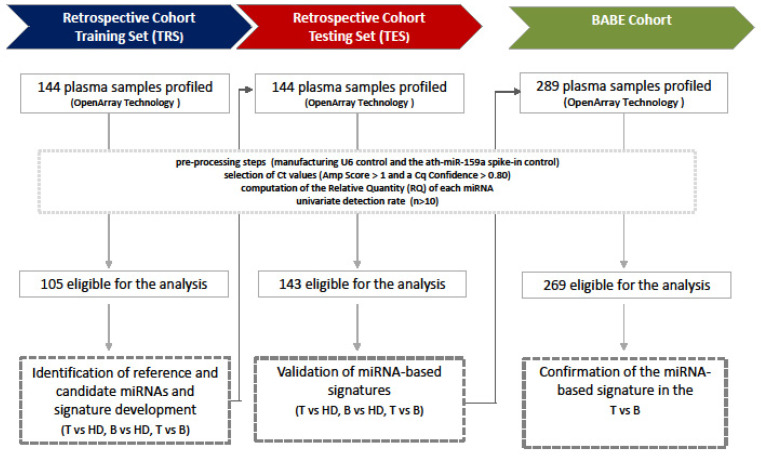
Study workflow. Graphical representation of plasma samples profiled and analyzed by OpenArray technology for each cohort.

Two independent cohorts of plasma samples were retrospectively (Retrospective Cohort) and prospectively (BABE—BreAst Blood Early diagnosis) collected at Fondazione IRCCS Istituto Nazionale dei Tumori di Milano (INT) between 2013 and 2017 (Figure 1). 

For the Retrospective Cohort, a total of 288 plasma samples were collected between 2013 and 2015, stored in the Biobank of INT, and randomly split into a training (TRS) and testing (TES) set by annotated disease status. Overall, Retrospective Cohort consisted of 99 healthy donor women (HD, 50 in the TRS and 49 in the TES), 100 patients with a breast tumor (T, 50 in the TRS and 50 in the TES), and 89 patients with a benign breast lesion (B, 44 in the TRS and 45 in the TES). Two-hundred eighty-nine plasma samples from the BABE study were prospectively collected between 2015 and 2017 from women with no previous diagnosis of cancer. These women underwent a biopsy to determine whether abnormal areas, identified by breast ultrasonography [maximum diameter of 20 mm (BI-RADS 4-5)], were malignant or benign lesions. Plasma samples were also collected at 12 ± 3 months of follow-up from 29 women at INT after being diagnosed with a malignant lesion (BABE-FU Cohort). Institutional approval from our independent ethics committee was obtained for this study (approval numbers INT111-13, INT144-14, and INT66-15). Patients gave informed consent to the use of their samples. All procedures were conducted per the Declaration of Helsinki. 

Table 1 summarizes the clinicopathological characteristics of the tumors in the cohorts by WHO classification [29]. The median age was 50 years (interquantile range, IQR: 42–56), 47 (IQR: 41–53), and 59 (IQR: 49–72) for HD, B, and T in the Retrospective Cohort, respectively; similarly, in the BABE Cohort, the median age was 46 (IQR: 41–52) and 55 (IQR: 48–70) for B and T, respectively. In the BABE-FU Cohort, the median age at surgery was 56 (IQR: 50–72). With regard to histology, benign breast disease was represented primarily by fibroadenoma (26%) and benign epithelial proliferations (47%) in the Retrospective Cohort and by fibroadenoma (35%) and benign epithelial proliferations (51%) in the BABE Cohort. All 29 BC patients of the BABE-FU Cohort received radiotherapy, 19 patients received hormone therapy alone and 5 patients received chemotherapy in addition to hormone therapy. 

### 2.2. Blood Collection, Plasma Separation, and RNA Extraction 

Blood was withdrawn before surgery from patients with T or B (Retrospective Cohort) in collaboration with the INT Biobank and before a core biopsy from women with imaging that was suggestive of breast cancer (BABE Cohort). For BABE patients in follow-up, blood was taken before surgery (T0) and 12 ± 3 months after surgery (T1) during a scheduled clinical evaluation. Blood was obtained from HDs at the time of blood donation in the Immunohematology and Transfusion Medicine Service of INT. Whole blood was collected in commercially available EDTA-treated tubes and centrifuged at 2200× *g* for 20 min at 4 °C to remove cells, and the recovered plasma was frozen immediately at −80 °C. Total RNA was extracted from 200 µL of plasma using the mirVana PARIS Kit, catalog number AM 1556, (Thermo Fisher Scientific, Waltham, MA, USA) according to the manufacturer’s protocol and eluted in 50 µL of buffer. 

To determine the influence of hemolysis on miRNA expression, an ad hoc forced hemolysis experiment was implemented. Hemolysis was artificially introduced into the plasma sample from an HD of the TRS by adding serial 1:4 dilutions of red blood cells (0.004–0.25% *v*/*v*) and uncontaminated plasma. Each sample was analyzed in triplicate over the entire absorbance spectrum on a NanoDrop™ 1000 (Thermo Fisher Scientific) before RNA extraction to check that hemolysis had occurred. The samples were then profiled for miRNA expression using OpenArray and analyzed per the simultaneous confidence intervals approach [30]. 

### 2.3. miRNA Profile

The miRNA in each sample was profiled by qRT-PCR using the OpenArray Human microRNA panel (OA), catalog number 4,470,189 (ThermoFisher Scientific), a fixed-content panel that contains 754 validated human TaqMan miR assays that were designed in miRBase, RRID:SCR_003152 v14.0. Briefly, the miRNAs in each sample were amplified with the manufacturer’s replicates of internal controls, including U6 and ath-miR-159a spike-in. Reverse-transcription and preamplification were performed using Megaplex RT Primers Human Pools A (v2.1) and B (v3.0), catalog number 4,444,750, per the manufacturer’s instructions. The samples, master mix, and Taqman reactions were arranged in a 384-well plate and transferred automatically to OpenArray plates using a QuantStudio OpenArray AccuFill System. The loaded OpenArray plate was sealed immediately, filled with OpenArray Immersion Fluid, and sealed by inserting the OpenArray Plug into the loading port. qRT-pCR was performed on a QuantStudio 12K Flex (Thermo Fisher). Primary data were retrieved using QuantStudio 12K Flex, v1.2.3.

### 2.4. Statistical Analysis

#### 2.4.1. Preprocessing Step 

For all cohorts, quality control of the data was performed to identify critical samples. The number of wells with a low ROX signal (ROX < 1000) and the number of detected miRNAs (Amp Score > 1 and Cq Confidence > 0.80) were evaluated for each sample. Outliers were flagged using the Hampel filter (values outside of the interval between the median of the distribution ±3 × the median absolute deviation were considered outliers). A hierarchical clustering of the correlation of expression profiles for all possible pairs of samples was also performed to assess the homogeneity of the data. Samples with detectable U6 manufacturing control and ath-miR-159a spike-in were included in the subsequent statistical analysis workflow. Ct values were analyzed in terms of Amp Score and Cq Confidence: only Ct values with Amp Score > 1 and Cq Confidence > 0.80 were considered in the subsequent statistical analysis. 

TRS preprocessed data were analyzed to identify a subset of reference miRNAs and a set of candidate miRNAs that were to be combined into signatures. Reference miRNAs were identified by running an updated version of the NqA R-function [31,32]. The relative quantity (RQ) of each miRNA, expressed on a logarithmic scale (log_2_RQ = −dCt), was then considered to be the pivotal variable for the subsequent statistical analysis. The same normalization was then used to analyze the TES and BABE data. 

#### 2.4.2. Retrospective Cohort Analysis

Two disease-specific comparisons were first considered for Retrospective Cohort: patients with breast tumor vs. healthy donors (T vs. HD) and patients with benign breast lesion vs. healthy donors (B vs. HD). In this step for the TRS data, only miRNAs that were detected in at least 10 subjects/disease were considered for the univariate analysis [33]. Differentially expressed miRNAs were identified in the univariate analysis by a nonparametric Kruskal–Wallis test. Candidate hemolysis-free miRNAs (according to the forced hemolysis experiment) that showed specific-disease statistical significance in the T vs. HD or B vs. HD comparison, but not both, were selected for the multivariate analysis. According to the required number of events per variable (EPV) [34], a standard method or the Penalized Maximum Likelihood Estimation (PMLE) approach [35,36] was used to combine significant miRNAs by multivariate analysis (i.e., all subset analyses). For each fitted model, the area under the receiver operating characteristic (ROC) curve (AUC) and its corresponding 95% confidence interval (95% CI) were calculated. Signatures that showed significant performance on the TRS, in terms of AUC values (i.e., lower 95% CI > 0.50), in the T vs. HD comparison but not in B vs. HD and vice versa were then evaluated on the TES data. Signatures that retained their significance on the TES were examined further in between T vs. B) by applying the same regression coefficients as in the TRS [33], to mimic the application to the subsequent BABE Cohort. 

#### 2.4.3. BABE Cohort Analysis

The most promising signatures in the Retrospective Cohort with regard to the T vs. B comparison were assessed in the BABE plasma samples alone or as extended models that included the CA15-3 epitope of the large transmembrane glycoprotein MUC1, that was tested in heparin plasma samples on an automatic electrochemiluminescence immunoassay system, catalog number 03045838122 (Cobas 6000 e601, Roche Diagnostics, Germany). The expression profiles of BABE-FU samples before surgery (T0) and 12 ± 3 months after surgery (T1) were compared by Wilcoxon signed rank (WSR) test for paired data.

All statistical analyses were carried out with SAS (Statistical Analysis System, RRID:SCR_008567, version 9.4.; SAS Institute, Inc., Cary, NC, USA) and R, adopting an α level of 5%. 

## 3. Results

### 3.1. Retrospective Cohort Analysis

Of the 144 TRS plasma samples that were profiled on the OpenArray plates, 105 (46 HD, 31 T, and 28 B) passed the preprocessing steps, and 255 miRNAs were considered in subsequent statistical analyses (Figure 1). By NqA 31, 4 miRNAs (hsa-miR-143-002249, hsa-miR-152-000475, hsa-miR-185-002271, hsa-miR-139-5p-002289) were identified for data normalization. Hemolysis-free miRNAs that were associated with disease status (T vs. HD or B vs. HD) were identified by univariate analysis by Kruskal–Wallis test. Specifically, 16 miRNAs (10 upregulated and 6 downregulated) were differentially expressed only in T vs. HD comparison, versus 14 (3 upregulated and 11 downregulated) only in the B vs. HD comparison (Table 2, Supplementary Figure S1). 

According to each comparison (T vs. HD or B vs. HD), candidate miRNAs were combined in multivariate manner (i.e., signatures) using the TRS data. In the T vs. HD scenario, 52 signatures retained their significant performance in the 143 samples that passed the preprocessing steps in the TES (i.e., T-promising signatures). No signatures showed significant performance in discriminating B vs. HD within the TES data. Among the 52 T-promising signatures, 26 had significant discriminatory performance in the T vs. B comparison for the TES. Supplementary Table S1 reports several descriptive statistics of the AUC values of these 26 signatures (i.e., TB-promising signatures) in the TRS and TES data. 

Among these 26 TB-promising signatures, 7 (M1–M7, top signatures) retained their significant performance in the TES even by applying the same regression coefficients that were obtained in the TRS (Table 3). 

**Figure 2:**
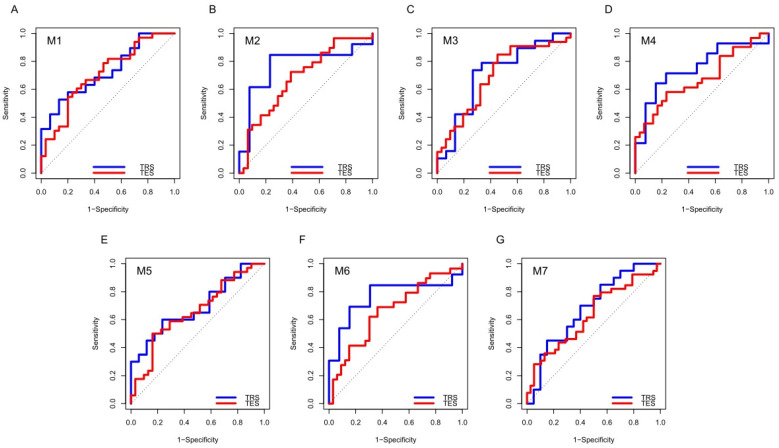
ROC curves of 7 miRNA-based signatures (M1–M7) in the TRS and TES sets. Each panel, (**A**): M1 signature; (**B**): M2 signature; (**C**): M3 signature; (**D**): M4 signature; (**E**): M5 signature; (**F**): M6 signature; (**G**): M7 signature, depicts the ROC curves of the 7 miRNA-based signatures in the training (TRS, in blue) and testing (TES, in red) sets of the Retrospective Cohort.

These top signatures were specific combinations of 5 miRNAs, from a maximum of 4 to a minimum of 2 miRNAs. Figure 2 reports the ROC curves of the top 7 signatures in the TRS and TES, with AUC values ranging from 0.680 to 0.769 and 0.632 to 0.708, respectively. 

### 3.2. BABE Cohort Analysis

**Figure 3:**
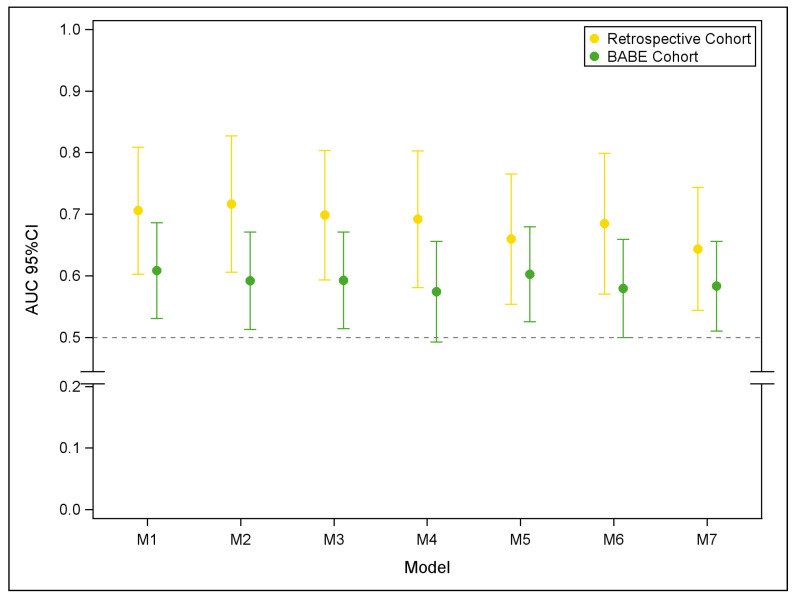
Performance of the 7-miRNA-based signatures (M1–M7) in the Retrospective and BABE Cohorts. Vertical bars representing 95% confidence interval of AUC values.

To confirm the relevance of all 7 signatures in a clinical setting, 289 EDTA-recovered plasma samples from the prospective BABE study and their technical controls were allocated and profiled in OpenArray plates, as performed for the TRS and TES data of the Retrospective Cohort. After the preprocessing steps, 269 samples (115 T and 154 B) were considered in subsequent statistical analyses. By fitting the M1-M7 signatures to the BABE data, the discriminatory performance of 5 signatures was confirmed (in terms of AUC value) but borderline significant for the remaining 2 (Figure 3).

A significant association (KW *p*-value: 0.036) between CA15-3 level and disease status was noted in the BABE Cohort, with higher levels in breast tumor samples (Supplementary Figure S2). However, when the analysis extended the 7 miRNA-based signatures (M1-M7) with CA15-3 levels, no significant increase in AUC values was observed (Supplementary Table S2), suggesting that an objective assessment of these candidate molecules could mitigate the limited diagnostic performance of currently available soluble markers.

### 3.3. BABE-FU Cohort Analysis

**Figure 4:**
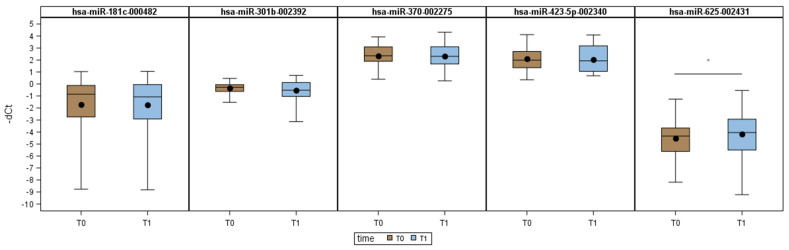
Evaluation of signature miRNAs at 2 time points. Distribution of each of the 5 miRNAs in signatures M1-M7 before surgery (T0) and 12 ± 3 months after surgery (T1). Each box indicates the 25th and 75th percentiles. The horizontal line in the box indicates the median, and the whiskers indicate the extremes. The black dot indicates the mean value. * if *p*-value < 0.05.

To examine the evolution of candidate miRNAs after surgical removal of the tumor, the circulating levels of the 5 miRNAs in signatures M1-M7 were measured in 29 BABE patients with a histological diagnosis of a tumor (Table 1), using matched plasma samples that were collected before surgery (T0) and 12 ± 3 months after surgery (T1). Their relative expression levels, according to the overall mean approach [37], were compared at the 2 time points. All patients were disease-free at T1. A significant increase in the log_2_(RQ) of miR-625 was observed after surgery (WSR test *p*-value: 0.044), but the 4 remaining miRNAs did not differ significantly at the 2 time points (Figure 4), suggesting that the origin of these 5 miRNAs was not from cancer cells.

## 4. Discussion

Although mammography remains the pillar diagnostic method in the early diagnosis of BC, current image-based approaches are associated with an increased frequency of biopsies to determine the malignant or benign nature of abnormal areas. Thus, reliable minimally invasive blood-based tests are long cherished to increase the compliance, while reducing cost, of population-based screens for BC. 

In this study, we analyzed circulating microRNAs in search of diagnostic biomarkers able to discriminate the benign and malignant nature of abnormal breast areas with imaging suggestive of BC (BI-RADS 4-5). We have identified 5 miRNAs that, when properly combined to form 7 miRNA-based signatures, can be applied to fluid biopsies to support diagnostic imaging. These results were obtained examining a retrospective cohort and confirmed in a prospective clinical cohort consecutively enrolled during the study. Using high-throughput OpenArray technology, over 700 microRNAs were analyzed in a retrospective cohort of plasma samples from age matched HDs and T or B patients, split into training and testing sets. Although distinct microRNAs emerged from the T vs. HD and B vs. HD comparisons in the TRS set, only signatures that discriminated between T and HD—not B and HD—were confirmed in the TES, highlighting the challenges of identifying circulating molecules that reflect the presence of benign breast disease. Nevertheless, out of 52 signatures distinguishing between T and HD, 26 significantly discriminated T from B lesions in the TES and, notably, 7 miRNA-based signatures comprising ad hoc combinations of 5 miRNAs retained significant performance even when the same regression coefficients obtained in the TRS was applied. Although the differences in blood samples from B patients were minimal with respect to HD, these results argue in favor of dissimilarities between malignant and benign breast blood samples that could be exploited in making a differential diagnosis. 

Consistently, 5 of the 7 combinations of 5 miRNAs maintained their ability to discriminate malignant from benign disease in our large BABE prospective cohort. These signatures were applicable to 93% of women with uncertain tumor or benign disease, indicating that the 5 constituent miRNAs are readily detected in plasma samples and that their absence (9 tumors, 11 benign lesions) is independent of disease status. Thus, it is conceivable that a small tumor, as in the early screening of the BABE Cohort, harbors circulating miRNAs that are sufficiently differentially expressed compared with benign breast disease. This is consistent with the detection of various circulating miRNAs, in a spontaneous model of mammary carcinogenesis, that are differently expressed from the non-transgenic siblings and that are maintained or differently represented along the stage of transformation [38]. 

Notably, one microRNA has a human homolog (has-miR-370) which belongs to the 7 signatures discriminating T from B patients. The miR-423-5p, detected at higher levels in T than B plasma samples, has been shown highly expressed in plasma and blood exosomes of breast cancer patients in comparison with healthy controls and significantly associated with clinical stage and Ki-67 levels [39]. The miR-625, which we found decreased in T versus B plasma samples, has been reported at lower levels in ductal lavage from patients with unilateral breast cancer versus ductal lavage of the contralateral normal breast [40]. Remarkably, the level of miR-625-5p increased after surgical removal of the tumor indicating its properness in combination with the other four miRNAs to form the diagnostic models for the presence of malignancies. In addition, this finding suggests that the source of miR-625-5p is not from neoplastic cells that rather negatively regulate its expression. Several datasets of microRNA expression in normal cell populations, show miR-625-5p expressed at high level in T lymphocytes (Supplementary Figure S3) and, therefore tumor cells might find benefit from lowering its expression in the attempt to escape immune surveillance. Although the presence of a minimal residual disease not detectable by conventional detection strategies cannot be excluded, the lack of change in the levels of miR-423-5p, miR-370-3p, miR-181c, and miR-301b after surgery also indicates their origin from cells other than the primary tumor. Accordingly, datasets of microRNA expression in normal cell populations [41,42,43] showed inflammatory cells, endothelial cells, fibroblasts and adipocytes as possible source of the 4 miRs. Specifically, the miR-423-5p (up in T vs. B plasma samples) was found enriched in immune populations, particularly B lymphocytes, compared with epithelium and endothelium (Supplementary Figure S3). Moreover, based on miRNA databases, in human plasma (PRJNA296772) and plasma-derived exosomes (PRJNA196121), miR-423-5p was among the top 20 most abundant circulating miRNAs [44]. Regarding the other 3 miRNAs, miR-301b-3p was higher in monocytes and endothelial cells; miR-181c-5p was expressed in T lymphocytes, along with miR-625-5p, and expressed in neutrophils and mast cells. Finally, miR-370-3p, was enriched in mesenchymal stem cells and mesenchymal-derived lineages, such as fibroblasts and adipocytes (including preadipocytes). Thus, no changes in the levels of miR-423-5p, miR-370-3p, miR-181c, and miR-301b after surgery could be indicative that inflammation and breast healing still occur at 1-year follow-up likely due to the adjuvant therapy and radiotherapy the patients received at T1 blood withdrawal.

Defining the biological meaning of circulating miRNAs proves to be particularly challenging due to their unknown origin and cell/tissue specific mechanism of action. Based on lists of predicted targets of the five miRNAs of interest, the most represented pathways are related to the control of cell cycle and senescence, but there is also the so called “Human T-cell leukemia virus 1 infection” KEGG pathway, which is implicated in chronic inflammatory diseases [45]. The literature provides data on validated targets, such as KRAS for miR-181c-5p [46], or SOX2 for miR-625-5p [47], which are important oncogenes in breast cancer.

Although the analysis of a defined set of miRNAs by qRT-PCR on the OpenArray platform might have overlooked additional or better-performing candidates in detecting BC, our signatures, obtained by properly combining miR-625, miR-423-5p, miR-370-3p, miR-181c, and miR-301b, significantly discriminated a tumor from a benign nodule, even in a prospectively recruited cohort of women with a high likelihood of malignancy, such as in the BABE Cohort. The collection of plasma samples from patients enrolled in the BABE-FU is still ongoing to evaluate possible time trends in the change of miRNA levels as well as to evaluate their potential prognostic values in predicting patients’ outcome. 

## 5. Conclusions

In this study, we identified and confirmed on a prospective clinical study five miRNAs-based signatures able to discriminate malignant from benign breast disease. Even though our signatures are unlikely to be used alone to make accurate BC predictions, our work supports the use of circulating miRNAs in distinguishing malignant from benign breast disease to complement imaging-based screens for the early diagnosis of BC and perhaps to spare unnecessary biopsies to a large fraction of women. Further studies are needed to confirm the analytically performance of our signature and to fully assess their clinical utility. To this end, an easy-to-use assay with the discovered miRNA signatures should be firstly developed and evaluated in the current clinical setting program on patients from the same target population.

## Figures and Tables

**Table 1:** Clinicopathological characteristics of breast cancer patients.

		Retrospective Cohort (*n* = 100)	BABE Cohort (*n* = 125)	BABE FU Cohort (*n* = 29)
		*n* (%)	*n* (%)	*n* (%)
Histology	IDC ^a^	74 (74)	87 (69)	17 (59)
	ILC ^b^	10 (10)	18 (15)	5 (18)
	IDC + ILC	5 (5)	1 (1)	1 (3)
	In situ ^c^	3 (3)	8 (6)	4 (14)
	IDC mixed ^d^	3 (3)	4 (3)	1 (3)
	Special Types ^e^	5 (1)	6 (5)	1 (3)
	Normal Tissue	-	1 (1)	-
IHC Histotype ^f^	Luminal A	17 (17)	34 (27)	12 (41)
	Luminal B	45 (45)	60 (48)	12 (41)
	Luminal HER2	11 (11)	5 (4)	-
	HER2	5 (5)	7 (6)	1 (3)
	Triple-Negative	19 (19)	5 (4)	-
	In situ	3 (3)	8 (6)	4 (15)
	Not determined	-	6 (5)	-
Grade	I	8 (8)	14 (11)	3 (11)
	II	43 (43)	72 (58)	21 (72)
	III	49 (49)	38 (30)	5 (17)
	Not determined	-	1 (1)	-
Tumor Size ^g^	T1	68 (68)	101 (81)	27 (93)
	T2	32 (32)	21 (17)	2 (7)
	Not determined	-	3 (2)	-
Lymph node	Negative	62 (62)	83 (66)	22 (76)
	Positive	38 (38)	24 (19)	7 (24)
	Not determined	-	18 (15)	-
ER ^h^	Positive	74 (74)	105 (84)	26 (90)
	Negative	26 (26)	14 (11)	3 (10)
	Not determined	-	6 (5)	-
PgR ^h^	Positive	63 (63)	95 (76)	19 (66)
	Negative	36 (36)	24 (19)	10 (34)
	Not determined	1 (1)	6 (5)	-
HER2 ^i^	Positive	17 (17)	14 (11)	2 (7)
	Negative	83 (83)	105 (84)	27 (93)
	Not determined	-	6 (5)	-
Ki-67 ^l^	Positive	79 (79)	79 (63)	13 (45)
	Negative	19 (19)	36 (29)	16 (55)
	Not determined	2 (2)	10 (8)	-
Age	Median (interquartile range)	59 (49–72)	55 (48–70)	56 (50–72)

^a^ IDC infiltrating ductal carcinoma; ^b^ ILC infiltrating lobular carcinoma; ^c^ Ductal in situ and intracystic tumor; ^d^ IDC plus mucinous or iperplasia or in situ; ^e^ Other invasive tumors: Apocrine, Tubular, Mucinous, Metaplastic and Papillary. ^f^ IHC Subtype: Luminal A: ER^+^, PgR^+or−^, Ki-67^−^, Luminal B: ER^+^, PgR^+or−^, Ki-67^+^, Luminal HER2: ER^+^, PgR^+or−^, HER2^+^, HER2: ER^−^, PgR^−^, HER2^+^, Triple-Negative: ER^−^, PgR^−^, HER2^−^; ^g^ T2 when size > 2 cm; ^h^ ER- and PgR-positive > 10% cell positivity by IHC; ^i^ HER2 positive scored 3+ by IHC or 2+/FISH-positive; ^l^ Ki-67-positive > 14% cell positivity by IHC.

**Table 2:** List of differentially expressed miRNAs in the two disease-specific comparisons in the TRS.

**T vs. HD**	**miRNA**	**#T**	**#HD**	**KW-*p* Value**	**Direction**
	hsa-miR-423-5p-002340	31	45	0.0003	up
	hsa-miR-21-000397	29	46	0.0006	up
	hsa-miR-148a-000470	30	46	0.0011	up
	hsa-miR-218-000521	31	42	0.0037	up
	dme-miR-7-000268	24	37	0.0046	up
	hsa-miR-324-3p-002161	31	45	0.0067	up
	hsa-miR-502-3p-002083	30	46	0.0067	up
	hsa-miR-625-002431	27	45	0.0081	down
	hsa-miR-18a-002422	31	46	0.0120	up
	hsa-miR-142-5p-002248	31	46	0.0127	down
	hsa-miR-301b-002392	21	43	0.0148	down
	hsa-miR-186-002285	31	46	0.0153	down
	hsa-miR-370-002275	16	43	0.0155	up
	hsa-miR-548c-5p-002429	20	35	0.0182	up
	hsa-miR-181c-000482	30	44	0.0190	down
	mmu-miR-134-001186	18	43	0.0237	down
**B vs. HD**	**miRNA**	**#B**	**#HD**	**KW-*p* Value**	**Direction**
	hsa-miR-128a-002216	26	45	0.0008	down
	hsa-miR-24-000402	27	46	0.0009	down
	hsa-miR-598-001988	26	45	0.0013	down
	hsa-miR-27a-000408	28	46	0.0027	down
	hsa-miR-133a-002246	27	46	0.0028	down
	hsa-miR-30c-000419	28	46	0.0048	down
	hsa-miR-320-002277	28	46	0.0051	up
	hsa-miR-148b-000471	27	46	0.0068	down
	hsa-miR-204-000508	27	45	0.0107	up
	hsa-miR-376a-000565	28	45	0.0126	down
	hsa-miR-331-000545	28	46	0.0133	down
	hsa-miR-324-5p-000539	27	46	0.0140	down
	hsa-miR-330-000544	24	42	0.0142	down
	hsa-miR-502-001109	15	27	0.0216	up

T: breast tumor, B: benign breast lesion, HD: healthy donor women, KW: Kruskal–Wallis Test.

**Table 3:** Performance of the 7 validated signatures (M1-M7) in the breast tumor vs. benign breast lesion.

Model	TRS Data AUC (95% CI)	TES Data AUC (95% CI)	*n*. miRNAs Included	miRNAs Included
M1	0.726 (0.556; 0.897)	0.708 (0.580; 0.837)	4	hsa-miR-423-5p-002340; hsa-miR-181c-000482; hsa-miR-625-002431; hsa-miR-301b-002392
M2	0.769 (0.562; 0.976)	0.683 (0.546; 0.820)	4	hsa-miR-423-5p-002340; hsa-miR-181c-000482; hsa-miR-301b-002392; hsa-miR-370-002275
M3	0.712 (0.527; 0.897)	0.696 (0.564; 0.828)	3	hsa-miR-181c-000482; hsa-miR-625-002431; hsa-miR-301b-002392
M4	0.753 (0.559; 0.946)	0.675 (0.539; 0.812)	3	hsa-miR-423-5p-002340; hsa-miR-625-002431; hsa-miR-370-002275
M5	0.688 (0.515; 0.861)	0.657 (0.522; 0.791)	3	hsa-miR-423-5p-002340; hsa-miR-625-002431; hsa-miR-301b-002392
M6	0.763 (0.557; 0.970)	0.660 (0.522; 0.799)	3	hsa-miR-181c-000482; hsa-miR-301b-002392; hsa-miR-370-002275
M7	0.680 (0.511; 0.849)	0.632 (0.507; 0.758)	2	hsa-miR-181c-000482; hsa-miR-301b-002392

TRS: training set; TES: testing set, AUC: area under the ROC Curve; CI: confidence interval.

## Data Availability

All data generated and analyzed during the current study are available from the corresponding author on reasonable request.

## Supplementary Materials

The following are available online at https://www.mdpi.com/article/10.3390/cancers13164028/s1, Figure S1: Distribution of expression levels of de-regulated miRNAs in the TRS. Panel A and B report the miRNAs distribution tumors vs. healthy donors comparison and in the benign lesions vs. healthy donors comparison, respectively. Each box indicates the 25th and 75th percentiles. The horizontal line inside the box indicates the median, and whiskers indicate the extreme measured values; Figure S2: Association between CA15.3 expression levels and disease status. Distribution of CA15.3 expression levels according to the disease status (malignant or benign lesions) of the BABE Cohort. Each box indicates the 25th and 75th percentiles. The horizontal line inside the box indicates the median, and the whiskers indicate the extreme measured values. Figure S3: In silico miRNA expression analysis in human primary cells. miR-423-5p, miR-181c-5p, miR-301b-3p, miR-625-5p and miR-370-3p expression levels in 26 human primary cells, comprising 19 blood cells, 6 stromal cells and mammary epithelial cells. Data are expressed as average reads per million miRNA reads (RPM) (± SD) and are not otherwise normalized. Sample number (*n*) for each cell type is indicated below expression bars; Table S1: Descriptive statistics (in terms of AUC) of the 26 TB-promising signatures; Table S2: AUC values and their corresponding 95% CI for each signature alone or with CA15.3 in the model.
