# Supplementary material for: Circulating miRNAs as Novel Non-Invasive Biomarkers to Aid the Early Diagnosis of Suspicious Breast Lesions for Which Biopsy Is Recommended
Source: Cancers (Basel). 2021 Aug 10;13(16):4028. doi: 10.3390/cancers13164028 (PMC8391908; doi:10.3390/cancers13164028)
Supplement: Supplementary file 1 [file cancers-13-04028-s001.zip › cancers-1323282-supplementary.pdf]

Supplementary Tables

Table S1. *Descriptive statistics (in terms of AUC) of the 26 TB-promising signatures.*

**n. of models**	**n. of miRNAs included in the model**	**TRS data** median (range)	**TES data** median (range)
3	5	0.809 (0.785-0.821)	0.703 (0.698-0.745)
7	4	0.770 (0.726-0.805)	0.710 (0.656-0.729)
13	3	0.775 (0.688-0.889)	0.673 (0.645-0.805)
3	2	0.730 (0.680-0.800)	0.638 (0.631-0.688)
**26**		**0.780 (0.680-0.889)**	**0.697 (0.631-0.805)**

Table S2. *AUC values and their corresponding 95% CI for each signature alone or with CA15.3 in the model.*

**Model**	**AUC**	**95% CI**
M1 + CA 15.3	0.619	(0.541;0.697)
M1	0.608	(0.531;0.686)
M2 + CA 15.3	0.619	(0.540;0.697)
M2	0.592	(0.513;0.671)
M3 + CA 15.3	0.613	(0.535;0.691)
M3	0.593	(0.515;0.671)
M4 + CA 15.3	0.584	(0.503;0.666)
M4	0.574	(0.492;0.656)
M5 + CA 15.3	0.607	(0.530;0.684)
M5	0.603	(0.525;0.680)
M6 + CA 15.3	0.618	(0.539;0.697)
M6	0.580	(0.500;0.660)
M7 + CA 15.3	0.614	(0.542;0.687)
M7	0.583	(0.510;0.656)

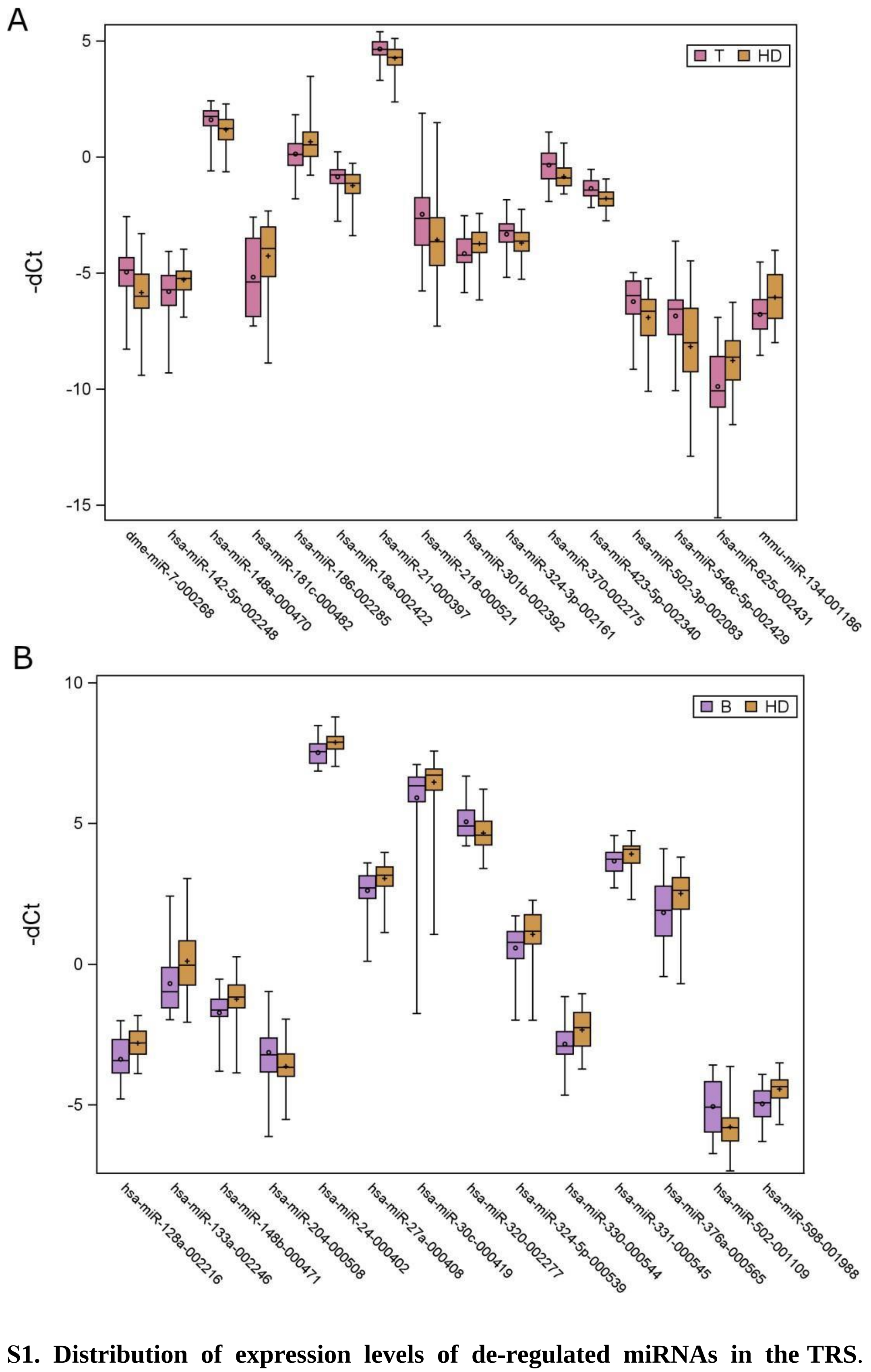

Figure S1. Distribution of expression levels of de-regulated miRNAs in the TRS. Panel A and B report the miRNAs distribution tumors vs healthy donors comparison and in the benign lesions *vs* healthy donors comparison, respectively. Each box indicates the 25th and 75th percentiles. The horizontal line inside the box indicates the median, and whiskers indicate the extreme measured values.

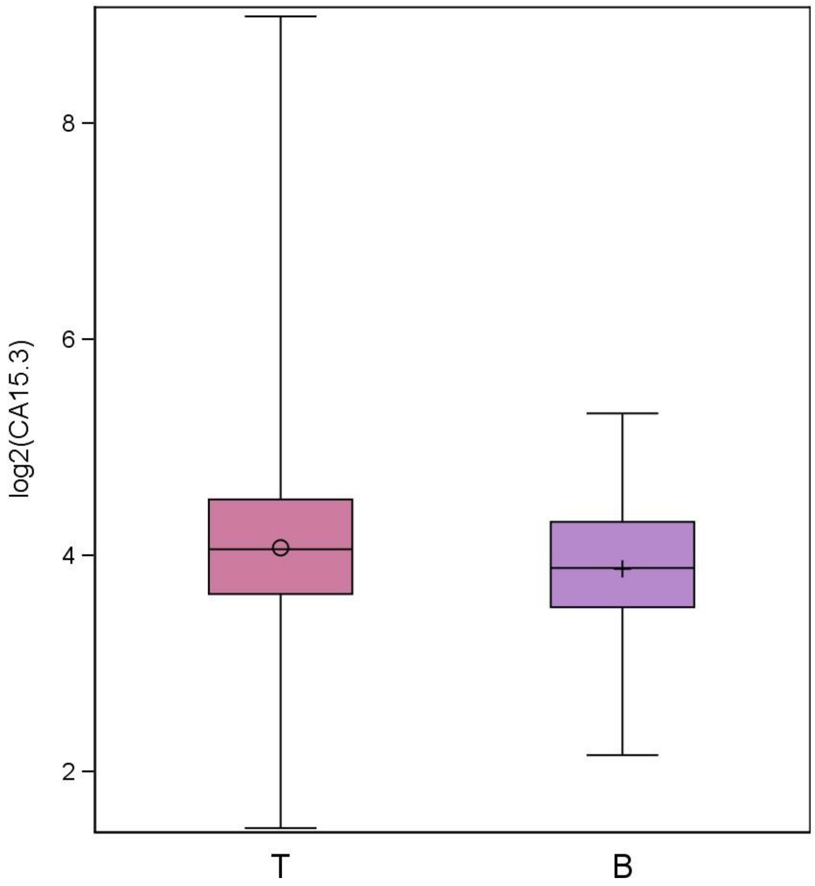

Figure S2. Association between C15.3 expression levels and disease status. Distribution of CA15.3 expression levels according to the disease status (malignant or benign lesions) of the BABE cohort. Each box indicates the 25th and 75th percentiles. The horizontal line inside the box indicates the median, and the whiskers indicate the extreme measured values.

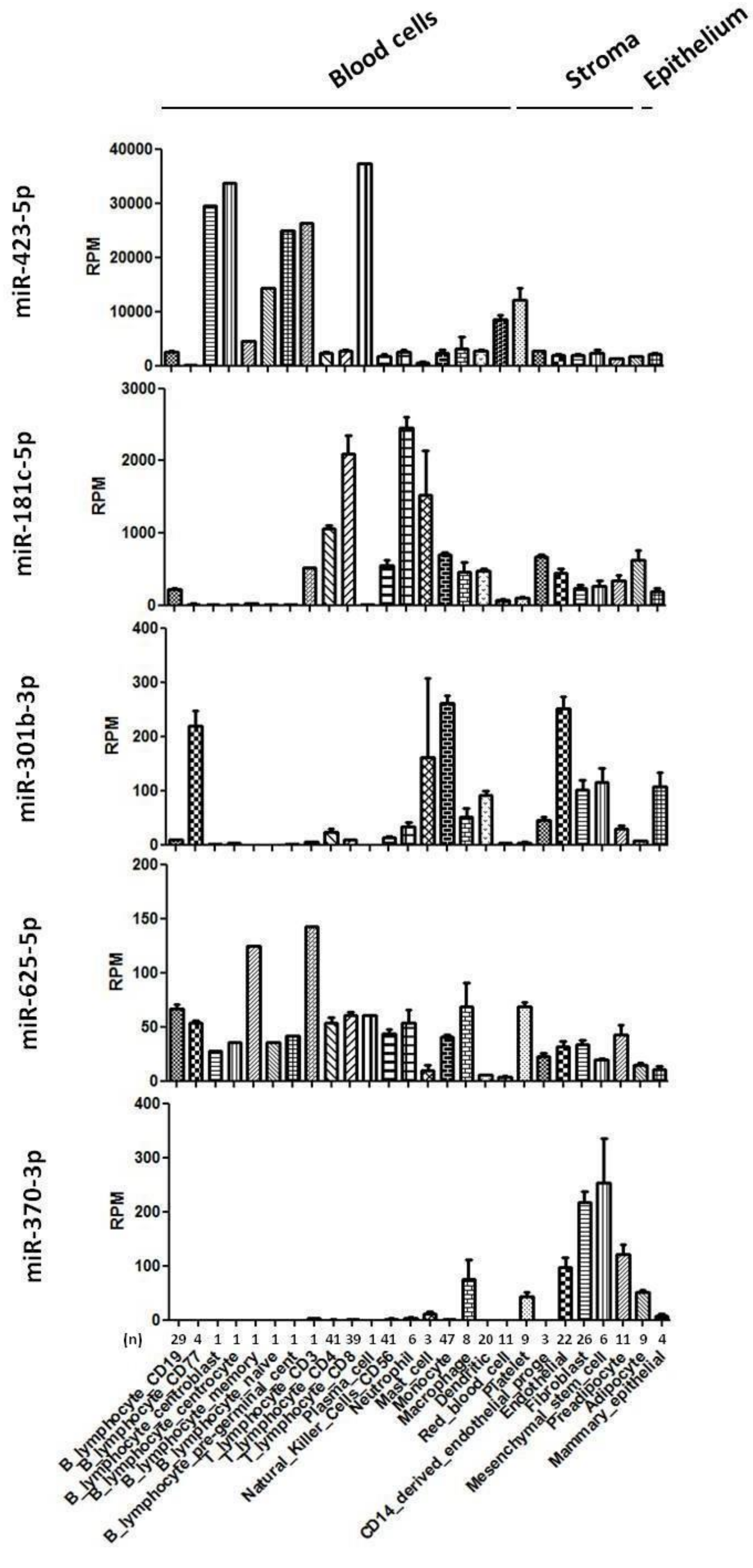

Figure S3. *In silico* miRNA expression analysis in human primary cells. miR- 423-5p, miR-181c-5p, miR-301b-3p, miR-625-5p and miR-370-3p expression levels in 26 human primary cells, comprising 19 blood cells, 6 stromal cells and mammary epithelial cells. Data are expressed as average reads per million miRNA reads (RPM) (± SD) and are not otherwise normalized. Sample number (n) for each cell type is indicated below expression bars.
